# Cambridgeshire Lifespan Autism Spectrum Service Clinic: Managing Demand, Capacity and Flow of Referrals for Adult Autism Assessment

**DOI:** 10.1192/bjo.2024.423

**Published:** 2024-08-01

**Authors:** Janine Robinson, Jasmine Taylor, Mark Squire, Andrea Woods, Irene James

**Affiliations:** Cambridgeshire Lifespan Autism Spectrum Service (CPFT NHS Trust), Cambridge, United Kingdom

## Abstract

**Aims:**

Referrals for adult autism assessment to the Cambridgeshire Lifespan Autism Spectrum Service (CLASS) have increased from 430 in 2019 to 887 in 2023, with demand exceeding capacity. The team enrolled in the Royal College of Psychiatrists’ Quality Improvement (QI) Demand, Capacity and Flow (DCF) Collaborative. The agreed aim was to increase the number of diagnostic assessments by 51% per month.

**Methods:**

Participants included the CLASS multi-disciplinary team (MDT), referrers, the provider improvement advisor and an autistic adult. Using the NHS Quality Service Improvement and Redesign (QSIR) six-step approach, a process map identified five key stages of the CLASS pathway. A project driver diagram was then used to identify change ideas in the referral, screening, pre-assessment, assessment and post-diagnostic stages.

Change ideas in the screening and assessment stages were prioritised and two Plan-Do-Study-Act (PDSA) cycles designed: PDSA 1) To reduce screening time by removing the first screening of referrals; PDSA 2) To increase the number of assessments conducted and completed in a single face-to-face appointment.

Data collected for PDSA 1 included: number of working days from date of referral to date added to waiting list and total screening time (minutes) per referral. Data were compared in a sample of 133 referrals from the two-stage screening process and 68 referrals from the one-stage process. Data collected for PDSA 2 included: average assessment time (minutes), average duration of open assessments, and the number of assessments completed within the same month. The data at Time 1 (before introducing PDSA 2) were compared with Time 2 (after PDSA 2) in a sample of 10 and nine referrals, respectively.

**Results:**

PDSA 1) Statistical Process Control (SPC) charts show a reduction in mean working days from 160 to 30 working days. The mean screening time per referral reduced from 33 minutes to 23 minutes. PDSA 2) SPC charts show that between Time 1 and Time 2 there was (i) a reduction in clinician time in minutes per assessment (m = 236.8 to m = 210), (ii) a reduction in working days assessment remained open (m = 39.4 to m = 6.4), (iii) a reduction in number of assessments involving multiple appointments (6 of 10 to 3 of 9), (iv) an increase in the number of assessments completed in the same month (3 of 10 to 7 of 9).

**Conclusion:**

These results show promise towards increasing DCF across the pathway, but further PDSAs (e.g., digitalising reporting, refining the post-diagnostic pathway) need to be implemented to achieve the overall aim.

